# Defining a glycemic persistence index (GPI) for continuous glucose monitoring

**DOI:** 10.21203/rs.3.rs-8841862/v1

**Published:** 2026-02-11

**Authors:** Ren Zhang

**Keywords:** Continuous glucose monitoring, CGM, glycemic persistence index, GPI, hyperglycemia

## Abstract

Chronic exposure to elevated glucose is a central feature of dysglycemia across the spectrum from prediabetes to diabetes. Continuous glucose monitoring (CGM) provides rich temporal glucose data, but effective summarization that integrates the magnitude and duration of sustained hyperglycemia into a single and parameter-free scalar remains challenging. We introduce the glycemic persistence index (GPI), a simple, threshold-free CGM-derived metric defined as the largest integer k such that at least k minutes are spent at glucose levels ≥ k mg/dL within a day. Geometrically, after ranking glucose values in decreasing order, GPI is given by the intersection at which glucose level and cumulative duration take the same value. Analysis of a public CGM dataset showed strong correlations between GPI and daily mean glucose and glucose variance, while substantial heterogeneity at fixed GPI values indicated that GPI captures complementary information beyond average exposure or overall variability. As a simple, device-independent, and threshold-free scalar, GPI quantifies hyperglycemia by jointly capturing its magnitude and duration, enabling consistent and intuitive glycemic profiling accessible to both specialists and non-specialists.

## Introduction

Diabetes and dysglycemia are highly prevalent worldwide, spanning a broad continuum from prediabetes to established diabetes and affecting an increasing proportion of the adult population [1, 2]. Across this spectrum, chronic exposure to elevated glucose represents a central feature of metabolic burden and long-term risk [3, 4].

Continuous glucose monitoring (CGM) has transformed the assessment of glycemic control by providing high-frequency, real-time glucose measurements across daily life. CGM is now widely used in individuals with diabetes and is increasingly applied in prediabetes and research settings, generating detailed temporal profiles of glucose exposure that extend well beyond what can be captured by sparse laboratory measurements [5, 6]. These data capture rich temporal structure, including fluctuations, excursions, and prolonged periods of dysglycemia, but their volume and complexity make direct interpretation challenging. As a result, effective summarization of CGM data into concise, interpretable daily metrics has become an essential task for both clinical and research applications. Commonly used CGM-derived summaries include daily mean glucose, measures of glycemic variability, and threshold-based metrics such as time spent outside predefined glucose ranges. These metrics capture important and complementary aspects of glycemic profiles and are widely used in both clinical practice and research [7–11].

Sustained exposure to elevated glucose over extended periods is conceptually distinct from brief excursions or overall dispersion and carries intuitive relevance across the spectrum of dysglycemia. Capturing this notion of persistence requires accounting for both the magnitude of glucose elevation and the duration over which elevated levels are maintained. An effective CGM summary metric that emphasizes persistence should integrate glucose magnitude and duration into a single, interpretable scalar, while remaining threshold-free, minimally assumption-driven, and comparable across devices and study settings.

To address these challenges, we introduce the glycemic persistence index (GPI), a simple, nonparametric metric derived from CGM data that condenses glucose magnitude and duration into a single scalar. GPI is intuitive and immediately interpretable by both specialists and non-specialists. By design, GPI does not rely on predefined glucose thresholds or distributional assumptions, enabling consistent application across CGM devices, sampling frequencies, and study settings. We examine the relationship between GPI and commonly used daily CGM summaries, including mean glucose and glycemic variability, to clarify how GPI relates to existing measures.

## Materials and methods

### Continuous glucose monitoring data

Continuous glucose monitoring (CGM) data were obtained from the publicly available BIG IDEAs Lab Glycemic Wearable Dataset hosted on PhysioNet [12, 13]. The dataset includes CGM recordings from 16 individuals collected using Dexcom CGM devices, with glucose values reported in mg/dL at 5-minute intervals. For each subject, CGM data were organized by calendar day. Days with incomplete recordings were excluded to ensure reliable daily summaries. Analyses were performed at the subject-day level using quality-controlled CGM records.

### Definition of the glycemic persistence index (GPI)

For each subject-day, CGM measurements were used to compute the GPI. Glucose values within a day were represented as a time series with corresponding timestamps and ranked in decreasing order of glucose concentration. Cumulative time was computed based on the native sampling interval, yielding the total duration (in minutes) associated with glucose values exceeding a given level.

Let *G*_*t*_ denote the glucose concentration (mg/dL) measured at time *t*, and let Δ *t*_*t*_ denote the duration (in minutes) represented by that CGM measurement, determined by the native sampling interval. The GPI was defined as

$$\text{GPI}=\text{max}\left\{k\in \mathbb{N}:\Sigma_{\left\{t:G_{t}\geq k\right\}}\Delta t_{t}\geq k\right\}.$$

This definition identifies the largest integer value *k* for which the cumulative time spent at glucose values greater than or equal to *k* mg/dL is at least *k* minutes. Thus, GPI jointly encodes glucose magnitude and duration on a common numerical scale, increasing when higher glucose levels persist for longer periods. GPI can be computed by iterating over integer glucose levels k, summing CGM durations at or above k, and identifying the maximum k satisfying the persistence condition. GPI is expressed in units of time (minutes) and is evaluated over integer values of *k*, ensuring that it is independent of CGM sampling frequency and directly comparable across devices and study settings.

### Statistical analysis, software, and visualization

All analyses were performed at the subject-day level using quality-controlled CGM data. Daily mean glucose and daily glucose variance were computed using standard definitions based on all available CGM measurements within each 24-hour period. Associations between the GPI and daily glycemic summaries were assessed using Pearson correlation coefficients. Statistical significance was evaluated using two-sided tests, with p < 0.05 considered statistically significant. All data processing and statistical analyses were conducted using Python 3. Data manipulation was performed using standard scientific computing libraries, and figures were generated using matplotlib.

## Results and Discussion

### Geometric definition of the glycemic persistence index

**Figure 1:**
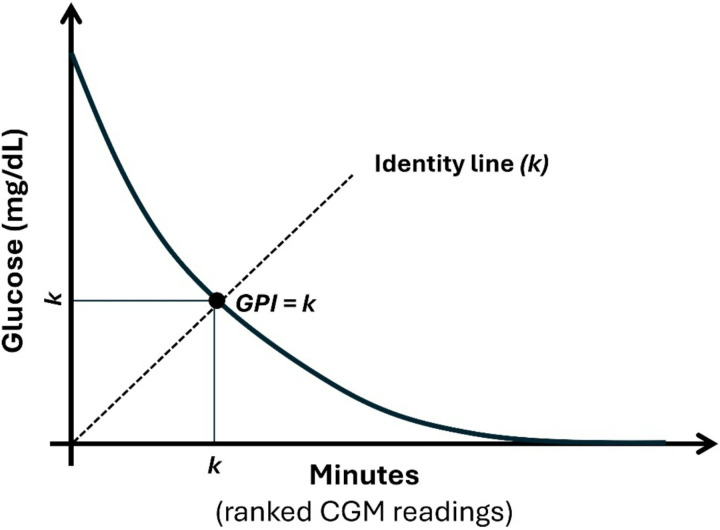
Geometric definition of the glycemic persistence index (GPI). Glucose measurements from a single day are ranked in descending order and plotted against time (minutes). The dashed identity line (k) represents the index-defining condition in which the same scalar k is applied to both duration and glucose magnitude. The GPI is defined as the largest value k for which at least k minutes exhibit glucose concentrations ≥ k mg/dL, corresponding to the intersection of the ranked glucose curve with the identity line.

We first illustrate the definition of the glycemic persistence index (GPI) using a geometric representation of ranked continuous glucose monitoring data (Fig. 1). For a given day, all glucose measurements are ranked in decreasing order and plotted as a function of cumulative time, producing a monotonically decreasing curve that summarizes both glucose magnitude and its temporal distribution. The diagonal identity line (y = x) serves as a reference that places glucose level and duration on a common numerical scale.

GPI is defined as the largest value k such that at least k minutes of the day exhibit glucose values ≥ k mg/dL. Graphically, this corresponds to the intersection between the ranked glucose curve and the identity line, with horizontal and vertical projections indicating the shared value k. This construction yields a single scalar that increases when higher glucose levels persist for longer durations, while remaining insensitive to brief excursions or isolated peaks. By jointly encoding severity and persistence in a unified geometric framework, GPI provides an interpretable summary of daily hyperglycemic burden. Larger GPI values reflect days characterized by sustained exposure to elevated glucose, whereas lower values indicate either shorter durations or lower magnitudes of hyperglycemia.

### Relationship of GPI with daily mean glucose and glycemic variability

**Figure 2:**
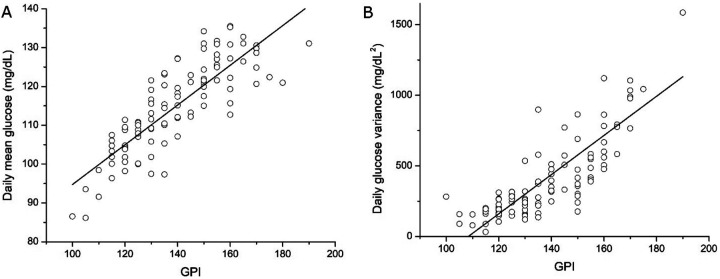
Relationship between GPI and daily mean glucose and variability. A) Relationship between GPI and daily mean glucose. The glycemic persistence index (GPI) was positively associated with daily mean glucose (Pearson correlation, r = 0.83, p < 0.001). Despite this strong association, substantial dispersion is observed at fixed GPI values, indicating that GPI captures aspects of glycemic persistence not fully reflected by average glucose alone. B) Relationship between GPI and daily glucose variance. The GPI was positively associated with daily glucose variance (Pearson correlation, r = 0.79, p < 0.001), indicating that greater glycemic persistence is accompanied by increased glycemic dispersion. Each point represents one subject-day from continuous glucose monitoring (CGM) data. The solid line denotes a least-squares linear fit used for visualization. Statistical significance was assessed using a two-sided Pearson correlation test.

We next evaluated how the GPI relates to commonly used daily summaries of CGM data, including daily mean glucose and glycemic variability (Fig. 2). Analyses were performed at the subject-day level using quality-controlled CGM records, allowing direct comparison of GPI with established metrics derived from the same daily glucose profiles.

GPI exhibited a strong positive association with daily mean glucose (Fig. 2A; Pearson correlation, r = 0.83, p < 0.001), indicating that days characterized by higher average glucose levels tend to show greater glycemic persistence as quantified by GPI. This relationship confirms that GPI is sensitive to overall glycemic exposure. Notably, however, substantial dispersion in daily mean glucose was observed across similar GPI values. This spread indicates that GPI is not a simple linear transformation of mean glucose, but rather reflects additional structure in the temporal distribution of elevated glucose values across the day.

We further examined the relationship between GPI and daily glucose variance as a measure of glycemic variability (Fig. 2B). GPI was also positively associated with daily glucose variance (Pearson correlation, r = 0.79, p < 0.001), consistent with the expectation that days with more persistent hyperglycemia are often accompanied by increased glucose fluctuations. As with mean glucose, considerable heterogeneity in glucose variance was evident at fixed GPI values, suggesting that GPI captures aspects of glycemic persistence that are related to, but not fully explained by, overall variability.

Taken together, these analyses show that GPI aligns with established daily glycemic summaries while remaining non-redundant with either average glucose or glycemic variability. By jointly reflecting the magnitude and duration of elevated glucose levels, GPI provides a complementary summary of daily glycemic burden that emphasizes sustained hyperglycemia rather than isolated excursions or global dispersion alone.

In this study, we introduce the glycemic persistence index (GPI), a simple and nonparametric summary metric derived from continuous glucose monitoring (CGM) data, and characterize its behavior using publicly available CGM recordings. Using a geometric framework, GPI condenses glucose magnitude and duration into a single scalar expressed in time units. We show that GPI is strongly associated with daily mean glucose and glycemic variability, while remaining non-redundant with either measure, indicating that GPI captures a distinct aspect of daily glycemic profiles.

Conceptually, GPI reflects the persistence of elevated glucose levels over time, integrating how high glucose rises and how long it remains elevated. This notion of persistence is distinct from brief excursions or overall dispersion and provides a complementary perspective on daily glycemic burden. By jointly encoding magnitude and duration, GPI emphasizes sustained hyperglycemia rather than isolated peaks, offering a concise representation of prolonged exposure to elevated glucose.

The relationships observed between GPI and commonly used CGM summaries further clarify its position among existing metrics. Daily mean glucose captures overall glycemic exposure, while measures of glycemic variability quantify dispersion around the mean. Threshold-based metrics, such as time spent outside predefined glucose ranges, summarize duration beyond fixed cutoffs. GPI differs from these approaches by integrating magnitude and duration into a single scalar without reliance on predefined thresholds or distributional assumptions. As such, GPI is best viewed as complementary to existing summaries, rather than as a replacement for established CGM metrics.

Several practical features of GPI may facilitate its use across research and clinical contexts. GPI yields a single scalar expressed in minutes, making it intuitive and immediately interpretable by both specialists and non-specialists. Its definition in continuous time renders it independent of device-specific sampling frequency and directly comparable across CGM platforms. Moreover, the absence of predefined glucose thresholds avoids arbitrariness associated with fixed cutoffs and supports consistent application across populations and study settings.

This study has several limitations. Analyses were performed using a limited number of subjects from a publicly available dataset, and CGM recordings spanned relatively short time periods. We did not examine associations between GPI and clinical outcomes, interventions, or long-term biomarkers, nor did we assess its performance across different disease states or treatment regimens. Accordingly, the present work is focused on defining and characterizing GPI, rather than establishing clinical utility or prognostic value.

Future studies may explore the application of GPI in larger and more diverse cohorts, including individuals with varying degrees of dysglycemia and across longer monitoring periods. Examining how GPI relates to clinical outcomes, treatment responses, and existing CGM reporting frameworks may further clarify its potential role in research and practice. In addition, integration of GPI into CGM analysis pipelines could facilitate broader evaluation and comparison with established glycemic summaries.

In summary, we introduce GPI as a threshold-free, device-independent CGM-derived metric that provides a concise summary of glycemic persistence by jointly capturing glucose magnitude and duration. By complementing existing CGM summaries, GPI offers an interpretable and flexible approach to characterizing sustained hyperglycemia and may serve as a useful addition to the repertoire of CGM-based glycemic metrics.

## Data Availability

The datasets generated and/or analyzed during the current study are available from the corresponding author upon reasonable request.
